# Hepatitis C virus may accelerate breast cancer progression by increasing mutant p53 and c-Myc oncoproteins circulating levels

**DOI:** 10.1007/s12282-023-01519-5

**Published:** 2023-11-16

**Authors:** Amira Fathy, Mohamed A. Abdelrazek, Abdelfattah M. Attallah, Amr Abouzid, Mohamed El-Far

**Affiliations:** 1 Research and Development Department, Biotechnology Research Center, New Damietta, Egypt; 2 Surgical Oncology Department, Mansoura Oncology Centre, Faculty of Medicine, Mansoura University, Mansoura, Egypt; 3 Biochemistry Division, Chemistry Department, Faculty of Science, Mansoura University, Mansoura, Egypt

**Keywords:** Breast cancer, Hepatitis C virus, p53, c-Myc, Progression

## Abstract

**Background:**

Hepatitis C virus (HCV) was reported to relate to polymorphous and frequent extrahepatic manifestation. Despite the limited studies, HCV viral oncoproteins may be implicated in breast cancer (BC) tumor aggressiveness. In a trial to elucidate a mechanistic link, this study aimed to investigate a mutant p53 and c-Myc oncoprotein expression levels in BC patients with and without HCV infection.

**Methods:**

A total of 215 BC patients (119 infected and 96 non-infected with HCV) were collected. ELISA was used for detection of anti-HCV antibodies, mutant p53, c-Myc, HCV-NS4, CEA, CA 125, and CA-15.3.

**Results:**

HCV infection was related to BC late stages, lymph-node invasion, distant metastasis, high grades, and large size. HCV-infected patients had a significantly (*P* < 0.05) higher WBCs, ALT and AST activity, bilirubin CEA, CA125 and CA15.3 levels, and reduced hemoglobin, albumin, and RBCs count. Regardless of tumor severity, HCV infection was associated with significant elevated levels of mutant p53 (22.5 ± 3.5 µg/mL; 1.9-fold increase) and c-Myc (21.4 ± 1.8 µg/mL; 1.5-fold increase). Among HCV-infected patients, elevated levels of p53 and c-Myc were significantly correlated with elevated tumor markers (CEA, CA 125, and CA15.3) and HCV-NS4 levels.

**Conclusions:**

This study concluded that HCV infection may be accompanied with BC severity behavior and this may be owing to elevated expression of mutant p53 and c-Myc oncoproteins.

## Introduction

Breast carcinoma (BC) is the most frequent tumor in women and one of the three most frequent malignancy worldwide (with about 2.3 million new patients in 2020) [1]. Although therapeutic and diagnostic improvements have prolonged BC patients overall survival, metastasis, and recurrence owing to other tumorigenesis factors, genetic signatures or molecular mechanisms are still critical challenges in BC treatment [2]. Thus, without fully understanding BC pathogenesis and mechanism related to the disease rapid progression, the treatment and prevention efficiency will always be limited [3].

To modern tumor research, viruses have been central and give profound insight into both non-infectious and infectious tumor causes and players in disease progression [4]. Among all viruses, nonhepatotropic and hepatotropic, HCV is described as the virus most often related to extrahepatic manifestations [5]. It is an oncovirus and an established hepatocellular carcinoma (HCC) risk factor [6]. In endemic HCV areas, the association of the virus with extrahepatic malignancy incidence was specifically observed [7]. The exact association between BC development and HCV infection remains elusive. However, recent nationwide population-based cohort studies in such endemic HCV areas, including large numbers of HCV-treated, -untreated, and -uninfected individuals, revealed that untreated HCV is potential BC risk factor [8]. HCV-untreated patients had the highest 9-year BC cumulative incidence [8]. Regarding disease progression, oncoproteins of HCV may involve in promoting some gene expression that, in turn, has been attributed to tumorigenesis [9]. In our previous study, we found that elevated HCV-NS4 in serum of BC patients was associated with BC severity features like infiltrated lymph nodes, high grades, advanced stages, and large tumor size [9].

The *p53* is the tumor suppressor gene that significantly correlated with several human cancers [10]. As a transcription factor, p53 protein promotes several cellular functions, such as energy metabolism, DNA repair, cell aging, cell cycle, cell differentiation, and apoptosis [10]. The destabilization, mutation, or inactivation of p53 is involved in BC progression and tumor cells proliferation [10, 11]. On another hand, *c-myc* is classical oncogene that may affect many tumors progression [12]. It regulates cell apoptosis, angiogenesis, differentiation, and proliferation [12, 13]. Many studies reported that c-Myc pathway is significantly promoted and enhanced BC progression [12, 14]. In a trial to elucidate some potential pathways involved in the association between HCV and BC progression, we performed this study to investigate a mutant p53 and c-Myc oncoproteins expression levels in BC patients with and without HCV infection. Also, we aimed to clarify whether these oncoproteins associate with tumor severity features and viral HCV-NS4 protein.

## Materials and methods

### Patients

This is a retrospective study included 215 Egyptian female patients with BC, aged 52.5 (± 13.5) years, who were recruited from Mansoura Oncology Centre, Mansoura University, Egypt. BC diagnosis was radiologically, clinically, and pathologically confirmed. All of patients did not have a history of any malignant neoplasms and cases with a history of other malignant tumors aside from BC are ineligible. Before any specific interventions and after informed consent, serum samples and clinical and pathological data were collected from all BC patients. The study protocol was approved by the Mansoura University Institutional Research Board (Research ID: MS.20.05.1130.R1.R2-2020/06/21) and was in accordance with Helsinki Declaration ethical guidelines (1975).

### Detection of HCV infection and laboratory assays

After blood withdrawal and within 2 h, serum samples were obtained by centrifugation (4000 rpm) and were stored at − 20 °C until use. BC patients’ sera were screened for anti-HCV antibody (ELISA; Biotech, UK) and some tumor-related biomarkers, including CEA, CA 125, and CA-15.3 (MyBioSource, San Diego, USA) using the 3rd-generation ELISA and based on the instructions of the manufacturers. Reactive samples were tested by a confirmatory RT-PCR test using a commercial HCV kit (Qiagen, Germany) and Real-Time PCR apparatus (Applied Bio-systems, USA). Also, according to Attallah et al. [9], HCV-infected patients were tested for HCV-NS4.

According to Attallah et al., all patients were also screened for both mutant p53 [15, 16] and c-Myc [15]. Briefly, diluted sera in carbonate/bicarbonate buffer (pH 9.6) coated microtiter plates (50 µl/well) and plates then were stand overnight at 4 °C for coating. After washing with PBS-T20 (0.05%; pH 7.2; 3 times), non-specific-binding sites were blocked using non-fat milk (0.2% (*w*/*v*) in carbonate/bicarbonate buffer; pH 9.6; 200 µl/well) and allowed for 1 h at room temperature. After washing, 50 µl/well of diluted anti-p53 or anti-c-Myc monospecific antibodies (Dokopatts) [1:100 in PBS-T20] were added and incubated with constant shaking for 2 h at 37 °C. After washing, plate wells were incubated with alkaline phosphatase labeled anti-mouse IgG (Sigma) diluted in 0.2% BSA in PBS-T20 for 1 h at 37 °C. For detection, the nitrophenyl phosphate substrate system (Sigma) was used. Absorbance was reading using at 405 nm (spectrophotometer; Metreiteck, Axiom, Burstadt, Germany).

### Immunohistochemical examination

BC specimens from BC patients were obtained from Mansoura Oncology Centre, Mansoura University, Egypt. Monoclonal antibodies for p53 and c-Myc (Santa Cruz Biotechnology, USA) were used and immunohistochemical analysis was blindly conducted. Intensity of p53 and c-Myc staining was quantified with the software Image-Pro Plus.

### Data analysis

All statistical measurements were done using computer programs GraphPad Prism (San Diego, CA) and IBM SPSS (IBM Corp, NY, USA) version 21 for Microsoft Windows. Different parameters were statistically described as frequencies, median, and range or mean ± standard deviation (± SD) when appropriate. Shapiro–Wilk test was used to evaluate the normal assumption of numerical data. Comparison between different data was assessed by Student’s *t *test or Mann–Whitney *U* test as appropriate. Also, Pearson’s two-tailed Chi-square (*Χ*^2^) test was used to compare different proportions. Two-sided *P* values < 0.05 were considered significant. Correlations were assessed by Pearson correlation coefficient.

## Results

### Clinicopathological characteristics

The patient-related clinical, hematological, and pathological data are summarized in Table 1. BC patients aged 52.5 (± 13.5) years. They had high levels of CEA, CA 125, and CA15. Patients were classified and staged according to the TNM staging system. Most of patients were had early stages (< T2; 66.5%), lymph-node invasion (77.2%), well-moderately differentiated tumor (G1–G2; 63.3%), and large tumor size (> 2 cm; 67.9%). Only 25.1% of patients had distant organ metastasis.

**Table 1:** Clinicopathological characteristics of patients (*n* = 215)

Variables	Value
Mean age (years)	52.5 ± 13.5
Alanine aminotransferase (U/L)	32.0 (26–42)
Aspartate aminotransferase (U/L)	20.0 (16.0–29.0)
Total bilirubin (mg/dL)	0.6 ± 0.1
Albumin (g/dL)	3.8 ± 0.6
Hemoglobin (g/dL)	12.2 ± 1.8
Red blood cells (× 10^12^/L)	4.5 ± 0.6
White blood cells (× 10^9^/L)	9.5 ± 0.8
Platelet count (× 10^9^/L)	259.4 ± 83.7
CEA (U/L)	28.0 (14.0–41.0)
CA 125 (U/L)	67.5 (47.5–134.3)
CA 15.3 (U/L)	34.2 (8.2–92.3)
Tumor stages	
Early stage (T1–T2)	143 (66.5%)
Late stage (T3–T4)	72 (33.5%)
Lymph-node invasion	
Negative (N0)	49 (22.8%)
Positive (N1)	166 (77.2%)
Metastasis	
Negative (M0)	161 (74.9%)
Positive (M1)	54 (25.1%)
Histological grade	
Low grade (G1–G2)	136 (63.3%)
High grade (G3)	79 (36.7%)
Tumor size (cm)	
≤ 2	69 (32.1%)
> 2	146 (67.9%)

Normally distributed data were expressed as mean ± standard deviation (SD), while non-normally distributed data were shown as median (interquartile range). Abbreviations: *CEA* carcinoembryonic antigen, *CA125* cancer antigen 125, *CA15.3* cancer antigen 15.3

### Impact of HCV infection on BC progression

According to HCV seropositivity in BC patients, there were 119 patients positive for HCV infection and 96 patients were negative for HCV. Interestingly, HCV infection was associated with BC progression (Table 2) as HCV-infected patients, compared to patients without infection, were more likely to be susceptible to sever tumor features including late tumor stages, lymph-node invasion, distant organ metastasis, high grades, and large tumor size.

**Table 2:** Impact of HCV infection on breast cancer progression

Categories	Breast cancer patients (*n* = 215)		*X*^*2*^; *P* value	Odd ratio (95% CI)
	HCV non-infected (*n* = 96)	HCV infected (*n* = 119)		
Tumor stage				
Early stage (T1–T2) (*n* = 143)	75 (52.4)	68 (47.6)	10.5; 0.001	2.7 (1.5–4.9)
Late stage (T3–T4) (*n* = 72)	21 (29.2)	51 (70.8)		
Lymph-node invasion				
Negative (N0) (*n* = 49)	28 (57.1)	21 (42.9)	4.1; 0.043	1.9 (1.0–3.7)
Present (N1) (*n* = 166)	68 (41.0)	98 (59.0)		
Metastasis				
Negative (M0) (*n* = 161)	79 (49.1)	82 (50.9)	5.1; 0.024	2.1 (1.1–4.0)
Present (M1) (*n* = 54)	17 (31.5)	37 (68.5)		
Tumor histological grade				
Low grade (G1–G2) (*n* = 136)	69 (50.7)	67 (49.3)	5.5; 0.023	2.0 (1.2–3.5)
High grade (G3) (*n* = 79)	27 (34.2)	52 (65.8)		
Tumor size				
≤ 2 cm (*n* = 69)	41 (59.4)	28 (40.6)	8.9; 0.003	2.4 (1.4–4.4)
> 2 cm (*n* = 146)	55 (37.7)	91 (62.3)		
Hb (g/dL)	11.9 ± 1.2	10.8 ± 1.3	0.0001	–
RBC (× 10^12^/L)	4.6 ± 0.5	4.1 ± 0.6	0.006	–
WBC (× 10^9^/L)	8.4 ± 2.5	11.6 ± 3.6	0.028	–
Platelet count (× 10^9^/L)	260.1 ± 78.7	234.7 ± 66.0	0.050	–
ALT (U/L)	32.0 ± 8.7	47.2 ± 14.6	0.002	–
AST (U/L)	23.5 ± 6.8	46.5 ± 12.8	0.001	–
Total bilirubin (mg/dL)	0.9 ± 0.3	1.3 ± 0.5	0.004	–
Albumin (g/dL)	3.8 ± 0.6	3.2 ± 0.3	0.003	–
CEA (U/L)	26.0 (10.1–32.8)	34.5 (17.1–46.8)	0.045	–
CA 125 (U/L)	49.5 (26.6–60.3)	109.5 (71.8–170.5)	0.0001	–
CA 15.3 (U/L)	32.8 (11.5–56.8)	76.5 (19.7–118.2)	0.0001	–

Normally distributed data were expressed as mean ± standard deviation (SD), while non-normally distributed data were shown as median (interquartile range). Abbreviations: *CEA* carcinoembryonic antigen, *CA125* cancer antigen 125, *CA15.3* cancer antigen 15.3

Also compared to non-infected patients, HCV-infected patients had a significantly higher count of white blood cells (WBCs), elevated liver enzymes activity and bilirubin levels, and reduced hemoglobin, albumin, and red blood cells (RBCs) count. Additionally, HCV infection was associated with higher level of tumor markers, including CEA, CA125, and CA15.3 (Table 2).

### HCV infection was associated with increased p53 and c-Myc

**Fig. 1:**
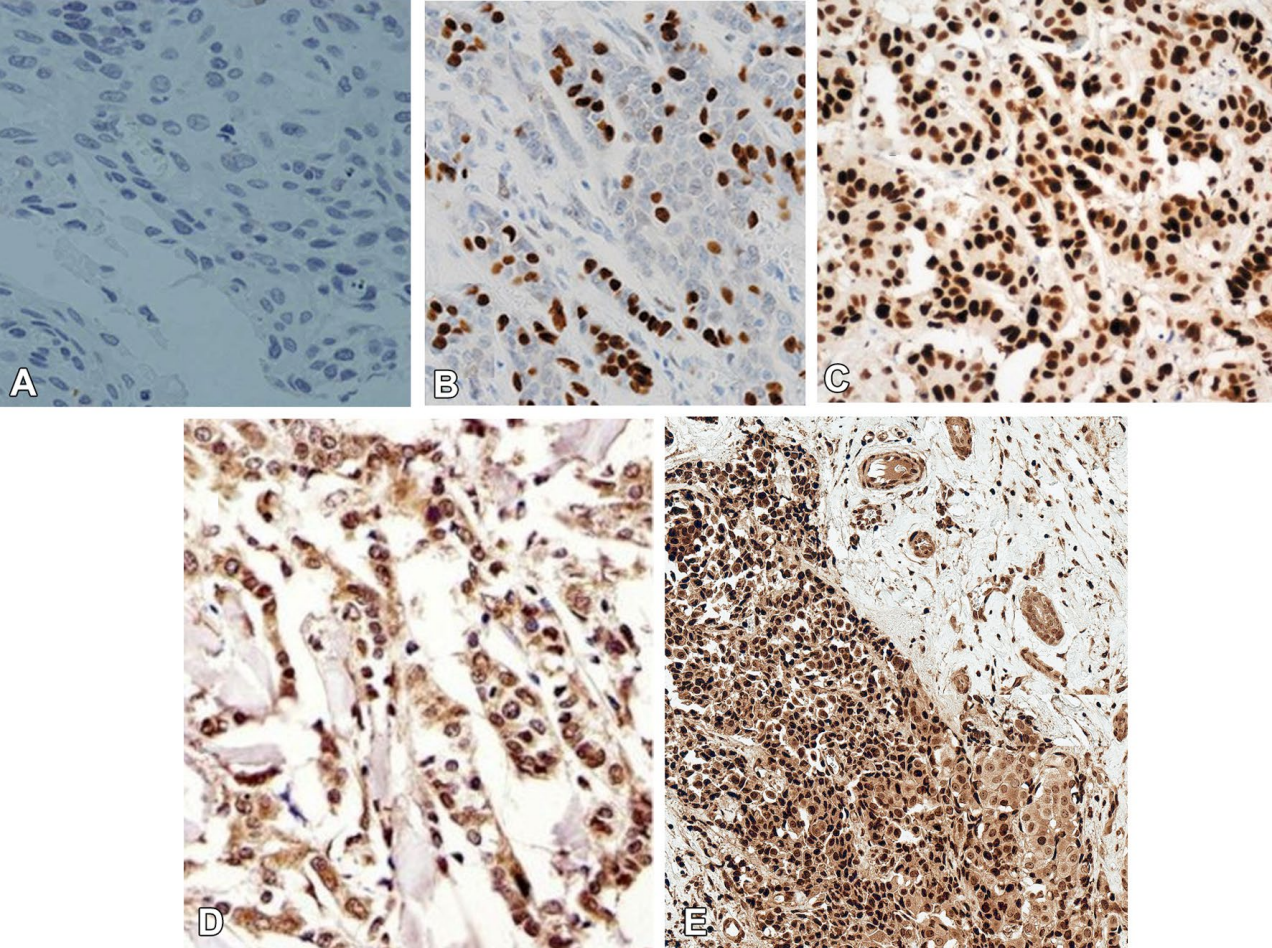
Immunohistochemical staining of p53 and c-Myc oncoproteins in BC tissues. Compared to (**A**) the negative staining, p53 expression was compared to (**B**) HCV-non-infected patients, significantly higher in (**C**) tissues from HCV-infected patients. Also, compared to (**D**) patients without HCV infection, c-Myc expression was significantly higher in (**E**) patients with HCV

**Fig. 2:**
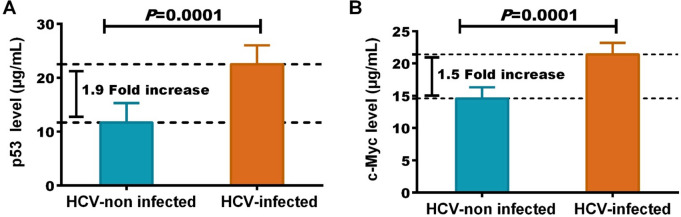
HCV infection was associated with elevated **A** mutant p53 and **B** c-Myc oncoproteins circulating levels

**Fig. 3:**
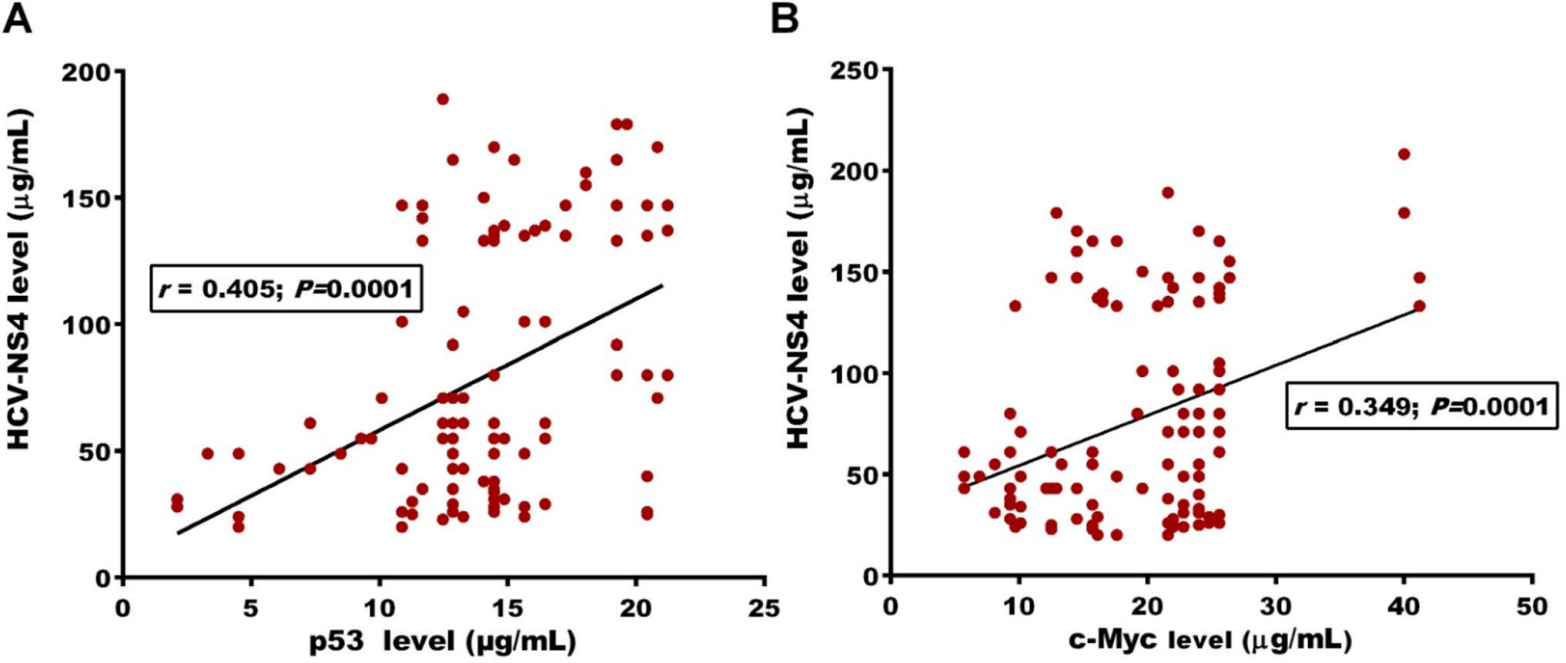
Elevated levels of HCV non-structural protein 4 were significantly correlated with **A** mutant p53 and **B** c-Myc oncoproteins’ circulating levels

IHC of BC tumor tissues revealed that the expression level of p53 (Fig. 1B, C) and c-Myc (Fig. 1D, E) was significantly higher in tissues from HCV-infected when compared with that of non-infected patients. HCV-infected BC women were associated with significant high levels of mutant p53 (22.5 ± 3.5; 1.9-fold increase) and c-Myc (21.4 ± 1.8; 1.5-fold increase) proteins than non-infected women (11.7 ± 3.6 and 14.6 ± 1.7, respectively), as shown in Fig. 2. Regardless of tumor severity, HCV infection was accompanied by a significant increase in p53 and c-Myc levels (Table 3). At the same tumor stage, BC women who infected with HCV had 1.4- and 1.7-fold increase in p53 and c-Myc levels, respectively compared to patients without infection. Similar results were obtained according to lymph-node invasion, distant metastasis, histological grade, and tumor size (Table 3). Among HCV-infected patients, there were positive correlations between elevated levels of p53 and c-Myc and other estimated parameters, including reduced platelets count and albumin levels and elevated tumor markers (CEA, CA 125, and CA15.3) (Table 4). Both mutant p53 (*r* = 0.405, *P* < 0.0001) and c-Myc (*r* = 0.349, *P* < 0.0001) were significantly correlated with HCV-NS4 levels in HCV-infected BC patients (Fig. 3).

**Table 3:** Distribution of p53 and c-Myc levels in BC patients according to HCV infection

Categories	p53 level (µg/mL)		*P* value	c-Myc level (µg/mL)		*P* value
	HCV non-infected (*n* = 96)	HCV infected (*n* = 119)		HCV non-infected (*n* = 96)	HCV infected (*n* = 119)	
Tumor stage						
Early stage (T1–T2)	9.9 ± 2.9	15.1 ± 3.1	0.001	14.8 ± 1.2	21.6 ± 1.3	0.028
Late stage (T3–T4)	13.6 ± 1.4	19.3 ± 2.6	0.030	13.9 ± 1.9	23.5 ± 1.2	0.015
Lymph-node invasion						
Negative (N0)	6.5 ± 1.1	14.5 ± 1.9	0.001	12.9 ± 1.5	17.7 ± 1.3	0.026
Positive (N1)	13.4 ± 1.9	20.5 ± 3.4	0.025	15.1 ± 1.2	22.2 ± 1.1	0.001
Metastasis						
Negative (M0)	10.3 ± 1.6	15.2 ± 1.5	0.034	13.3 ± 1.1	19.7 ± 1.8	0.007
Positive (M1)	14.9 ± 2.2	22.1 ± 2.5	0.039	14.2 ± 1.7	25.7 ± 2.1	0.019
Tumor histological grade						
Low grade (G1–G2)	9.6 ± 1.1	14.9 ± 2.3	0.027	14.1 ± 1.3	20.0 ± 1.3	0.024
High grade (G3)	13.7 ± 1.9	24.3 ± 3.1	0.032	14.5 ± 1.2	23.2 ± 1.3	0.009
Tumor size (cm)						
≤ 2	9.4 ± 1.3	14.1 ± 1.9	0.036	13.5 ± 1.3	22.6 ± 2.5	0.015
> 2	13.4 ± 1.5	21.6 ± 2.9	0.042	16.1 ± 1.2	21.4 ± 1.1	0.011

Data were expressed as mean ± standard deviation (SD). Significant difference was assessed by Student’s *t *test. *P* < 0.05 was significant

**Table 4:** Correlation between p53 and c-Myc levels and other estimated parameters in HCV-infected BC patients

Variables	p53 level (µg/mL)		c-Myc level (µg/mL)	
	Correlation (*r*)	*P* value	Correlation (*r*)	*P* value
Hb (g/dL)	− 0.162	0.477	− 0.160	0.062
RBC (× 10^12^/L)	− 0.168	0.053	− 0.045	0.598
WBC (× 10^9^/L)	0.227	0.770	0.046	0.614
Platelet count (× 10^9^/L)	0.241	0.005	0.238	0.006
ALT (U/L)	0.026	0.779	0.125	0.172
AST(U/L)	0.033	0.703	0.154	0.073
Total bilirubin (mg/dL)	0.045	0.609	0.095	0.271
Albumin (g/dL)	-0.226	0.010	-0.202	0.020
CEA (U/L)	0.264	0.011	0.241	0.036
CA 125 (U/L)	0.278	0.007	0.236	0.023
CA 15.3 (U/L)	0.368	0.001	0.433	0.0001

Correlation was assessed by Pearson correlation coefficient analysis in case of normally distributed data or Spearman's rank correlation coefficient analysis in case of non-normally distributed data.* P* < 0.05 was significant

Abbreviations: *Hb* hemoglobin, *RBC* red blood cell, *WBC* white blood cell, *ALT* alanine aminotransferase, *AST* aspartate aminotransferase, *CEA* carcinoembryonic antigen, *CA125* cancer antigen 125, *CA15.3* cancer antigen 15.3

## Discussion

However, HCV infection is associated with early onset BC [17] or not [6], and other important studies found an association between chronic HCV infection and BC severity [9]. In a previous study, although there was no association between HCV positivity in BC patients compared to patients with benign diseases (27.5 vs. 23.7%), our team found that HCV-NS4 was increased in BC HCV-infected patients compared to HCV-infected patients with benign diseases [9]. Elevated viral protein levels were related to tumor severity including infiltrated lymph-node high grades, late stages, and large size [9]. Here, we investigated the association between the impact of HCV infection on BC progression and mutant p53 and c-Myc oncoproteins’ expression.

First of all, HCV infection was more frequent in patients with tumor aggressiveness features including late stages (70.8 vs*.* 29.2%), lymph-node invasion (59 vs. 41%), distant metastasis (68.5 vs. 31.5%), high grades (65.8 vs. 34.2%), and large size (62.3 vs. 37.7%). Regarding clinical data, and compared to HCV non-infected patients, HCV-infected patients had significantly higher WBCs, ALT and AST activity and bilirubin levels, and reduced hemoglobin, albumin, and RBCs’ count. Moreover, HCV infection was associated with elevated CEA, CA125, and CA15.3 levels.

These results are in consistent with other limited reports that deal with the impact of HCV viral infection on BC severity. Some HCV-related viral proteins may promote the regulation of some gene expression that may be accompanied with transformation, apoptosis inhibition, and tumorigenesis [9, 18]. Elevated HCV-NS4 protein levels were significantly related to BC severity including advanced stages, high histological grades, large tumor size, and lymph-node invasion [9]. Regarding other BC clinically relevant aspects, other studies also may be in the same line with our findings were concerning with the impact of HCV infection on BC patients receiving chemotherapy. They found that HCV‐positive patients were associated with significantly high risk of longer time to complete treatment, dose delays, dose modifications, hematotoxicity-related dose delays, and hospitalization during chemotherapy [19].

Interestingly, compared to non-infected BC patients, we found that HCV-infected patients were associated with significant high levels of mutant p53 (1.9-fold increase) and c-Myc (1.5-fold increase) proteins. Regardless of tumor severity, these elevated levels of the two oncoproteins were associated with HCV infection. As, at the same tumor stage, BC women who infected with HCV had 1.4- and 1.7-fold increase in p53 and c-Myc levels, respectively, compared to patients without infection. The same was obtained regarding lymph-node invasion, distant metastasis, tumor grade, and tumor size. Moreover, the elevated levels of mutant p53 and c-Myc were positively correlated with elevated CEA, CA 125, and CA15.3 and also with HCV-NS4 [*r* = 0.405, *P* < 0.0001 for p53; *r* = 0.349, *P* < 0.0001 for c-Myc].

In HCV-related HCC, mutations of *P53* were common particularly in Africa [20, 21]. Moreover, *P53* nucleotide change were more frequent in HCV-related than in HBV-related HCC [21]. In BC, *P53* is the most commonly mutated gene. Its mutations inhibit its transcriptional activity and are greatly related to progression and poor survival in BC patients [22]. *P53* mutations was reported to be higher in high-grade and advanced-stage BC and these mutations have been related to BC with aggressive behavior like TNBC [23]. Besides inhibition of p53 functions, mutations in p53 can acquire oncogenic activity by gain-of-function (GOF) mechanisms that has been reported to promote genome instability [24]. Mutant p53 also can increase expression of chromatin-regulated genes which related to enhanced histone acetylation and methylation and contributes to BC progression [25]. Also, elevated mutant p53 levels are associated with many events that related to poor clinical outcomes and increased BC metastasis and invasion [22], including evading apoptosis [26] and activation of the transcription of many genes related to cell proliferation [27].

There was a mechanistic link between enhanced c-Myc expression and HCV infection [28]. In transgenic murine model expressing the entire HCV open-reading frame, elevated c-Myc expression was reported in vivo, suggesting a direct role of HCV protein expression in c-Myc induction. Through Akt activation and subsequent *β*-catenin stabilization, HCV non-structural proteins were reported to be responsible for c-Myc promoter activation [28]. In the majority of human tumors, *c-myc* oncogene is deregulated or amplified [29]. c-Myc drives several aspects of cancer metastasis and progression by promoting cell proliferation and survival, differentiation block, genetic instability, cell invasion, and migration [30]. In BC, c-Myc overexpression is frequent in invasive and high-grade tumors and is consistently related to early recurrence and poor outcome [31, 32]. In this context, c-Myc also promotes tumor-associated macrophages’ activation, which increase tumor’s aggressiveness [33]. Compared to HER2 and ER/PR + amplified BCs, c-Myc is disproportionately elevated in triple-negative BC (TNBC) [34]. Distant lethal BC metastases from c-Myc-unamplified primary cancers often gain c-Myc amplification [35]. Even when it is not overexpressed, the cancer microenvironment is maintained by c-Myc through inflammation, angiogenesis, and instructing tissue remodeling [36]. Inhibition of c-Myc was recently reported to stop progression of metastatic BC by blocking seeding, invasion, and growth [30].

In conclusion, this study findings concluded that HCV infection in BC patients may be accompanied with severity behavior of the tumor. These effects may be owing to the elevated expression of mutant p53 and c-Myc oncoproteins as HCV-infected BC patients were associated with elevated levels of these oncoproteins and subsequent severity features, including late stages, high histological grades, lymph-node invasion, and distant organ metastasis. Future large multicentre studies to confirm these results and investigate other mechanistic links between the concurrent HCV and BC are warranted. Also, other in vitro and in vivo studies are needed to perform experiment to clarify whether HCV infection causes increasing p53 and c-Myc proteins.

## Data Availability

Data of this study are available from the corresponding authors.
